# Media multitasking is associated with altered processing of incidental, irrelevant cues during person perception

**DOI:** 10.1186/s40359-018-0256-x

**Published:** 2018-10-11

**Authors:** Richard B. Lopez, Julia M. Salinger, Todd F. Heatherton, Dylan D. Wagner

**Affiliations:** 1 Department of Psychology, Rice University, 6500 Main Street, MS-663, Houston, TX 77030 USA; 2 Department of Psychology and Neuroscience, University of Colorado Boulder, Muenzinger D244, 345 UCB, Boulder, CO 80309 USA; 3 Department of Psychological and Brain Sciences, Dartmouth College, 6207 Moore Hall, Hanover, NH 03755 USA; 4 Department of Psychology, The Ohio State University, 140H Lazenby Hall, Columbus, OH 43220 USA

**Keywords:** Person perception, Media multitasking, Individual differences, Social cues

## Abstract

**Background:**

Media multitasking (MMT)—using and switching between unrelated forms of media—has been implicated in altered processing of extraneous stimuli, resulting in performance deficits. Here, we sought to extend our prior work to test the hypothesis that MMT might be associated with enhanced processing of incidental environmental cues during person perception.

**Method:**

We tested the relationship between individual differences in MMT and person perception, by experimentally manipulating the relevance of environmental cues that participants could use to make trait and personality judgements of an unfamiliar social target. Relevant environmental cues consisted of neat or messy arrangements of the target’s belongings, whereas irrelevant cues consisted of similarly neat or messy arrangements of the testing room in which participants viewed a video of the target.

**Results:**

In general, relevant cues affected ratings of the target’s conscientiousness. Additionally, and consistent with our hypothesis, there was a significant interaction between irrelevant cue condition and MMT, such that high media multitaskers more readily incorporated irrelevant environmental cues into their evaluations of the target’s conscientiousness.

**Conclusions:**

These results suggest that high media multitaskers are more responsive to irrelevant environmental cues, which in turn can lead them to form inaccurate impressions of others.

## Background

In the twenty-first century, humans face a unique cognitive challenge never faced before by our species: to divide attention between multiple media devices, such as smartphones, tablets, computers, and television. Because content across devices often competes for attention, many people attempt to engage in media multitasking (MMT), the simultaneous use of and switching between unrelated forms of media (e.g., tablet, smartphone, computer, smartwatch, etc.). Anyone who has ever looked up reviews or actor filmographies on their smartphone while binge watching a television show is familiar with this phenomenon. However, the human brain has limited attentional resources [1] and evolutionarily is ill-equipped to process this barrage of stimuli [2]. Under such conditions, irrelevant external cues may compete for people’s attention and become incorporated into their evaluations. Here, we examine the relation between MMT, irrelevant external cues, and social perception.

Previous research has associated MMT with incorporation of extraneous, irrelevant cues during cognitive tasks. In a landmark study researchers showed that high (versus low) media multitaskers were unable to filter extraneous cues in the environment while performing cognitive tasks, leading to the seemingly paradoxical finding of reduced performance in high media multitaskers on a task-switching task [3]. Additionally, high MMT may cause people to indiscriminately attend to peripheral cues, whether or not those cues are helpful and relevant to the task at hand [4]. Some have proposed that high media multitaskers have domain-general biases in attention [5]. For example, in research assessing how MMT is related to processing information, MMT was associated with increased responsiveness to rewarding, extraneous food cues and put people at higher risk for obesity [6, 7].

Here, we sought to extend our prior work to test the hypothesis that MMT might be associated with altered processing of incidental environmental cues during person perception. As an undeniably social species [8], human beings are motivated to make inferences about others’ traits and intentions, as well as to perform behaviors that balance needs for survival and sociability. Various environmental stimuli can influence how people are perceived, which in turn impact downstream social behaviors such as approach (or withdrawal), affiliation, and cooperation. Indeed, person perception can be influenced by incidental cues in the environment that need not be consciously perceived, yet nonetheless prime and shape behavior [9, 10].

One category of such cues is the material objects and decor in residential and personal settings, since objects and decorating schemes in one’s personal space often serve as statements of personality and identity [11, 12]. Moreover, as research shows, priming with mundane, common objects can subtly induce changes in the subsequent behavior of the perceiver [13]. Thus, it is likely that incorporation of incidental environmental cues into person evaluation may lead to attributional errors, especially if those cues are irrelevant or inaccurate indicators of the target’s personality traits. It is important, therefore, to identify person-environment interactions in which implicit incorporation of irrelevant cues is likely to occur.

In this study, we tested whether high media multitaskers would incorporate incidental environmental cues into their trait judgments of a social target. To this end, we adapted Gosling and colleagues’ “Room With a Cue” procedure, which examines how people use environmental cues found in offices and bedrooms to make trait judgments about previously unknown individuals [11]. Gosling’s work drew from the earlier perspective of Egon Brunswik (1952), who proposed that environmental cues in a living space can serve as a “lens” through which perceivers evaluate the personality dimensions of the space’s inhabitant(s) [14]. Brunswik also suggested that this process is complex because perceivers may use some cues more than others, and because cues can vary in the extent to which they reflect the occupant’s personality traits. That is, if the environment is a private space, it likely contains diagnostic cues that are indicative of the inhabitant’s personality and dispositions.

Here, participants watched a video clip of a previously unknown social target (being interviewed in his dorm room about his daily routine at college. Following the video, participants made trait judgments of the target’s conscientiousness. We focused on conscientiousness because the cues used for these judgments (i.e., those pertaining to order and tidiness) are visually salient and easy to manipulate in a lab setting, and also because participants in Gosling and colleagues’ study made accurate and reliable judgments along this trait dimension [11]. To alter the nature of the environment, we experimentally manipulated relevant and irrelevant cues that participants could incorporate into these trait judgments. Relevant cues consisted of objects in the video clip depicting the target’s personal room as neat or messy, whereas irrelevant cues consisted of neat or messy arrangements of objects in the testing room where participants watched the video clip. Participants’ tendency to media multitask was assessed via a brief questionnaire.

We hypothesized that, replicating previous work, participants would incorporate the relevant (video) cues into their judgments of the target’s conscientiousness (i.e., the neat or messy room conditions depicted in the video would bias participants to rate the target as more or less conscientiousness, respectively), with the possibility that high MMT would be associated with exaggerated effects on their ratings. Critically, we also predicted that high (versus low) media multitaskers would be more likely to incorporate *irrelevant* cues in their trait judgments. Specifically, we hypothesized that high media multitaskers would: (1) attribute lower conscientiousness to the target in the video in the messy testing room condition (regardless of the neat or messy room cues in the target’s depicted dorm room); and (2) give higher ratings of conscientiousness in the neat testing room condition (again, regardless of video condition).

## Method

### Participants

One hundred and three undergraduate students (65 females; Mean age = 18.75, *SD* = 1.00) were recruited to participate in the study in return for course credit, with the goal of having at least 20 participants per cell in our two-by-two experimental design. Sample sizes in the 25–30 range are sufficient to achieve about 80% statistical power to detect small to medium effects for either main effect or interaction terms [15]. Seven participants had incomplete data across one or more measures of interest and were thus excluded from the regression model detailed below. This resulted in a final sample size of 96 for all subsequent analysis (final N per cell = 24). Informed consent was obtained from all participants in accordance with guidelines set by the Committee for the Protection of Human Subjects’ at Dartmouth College.

### Procedure

**Fig. 1:**
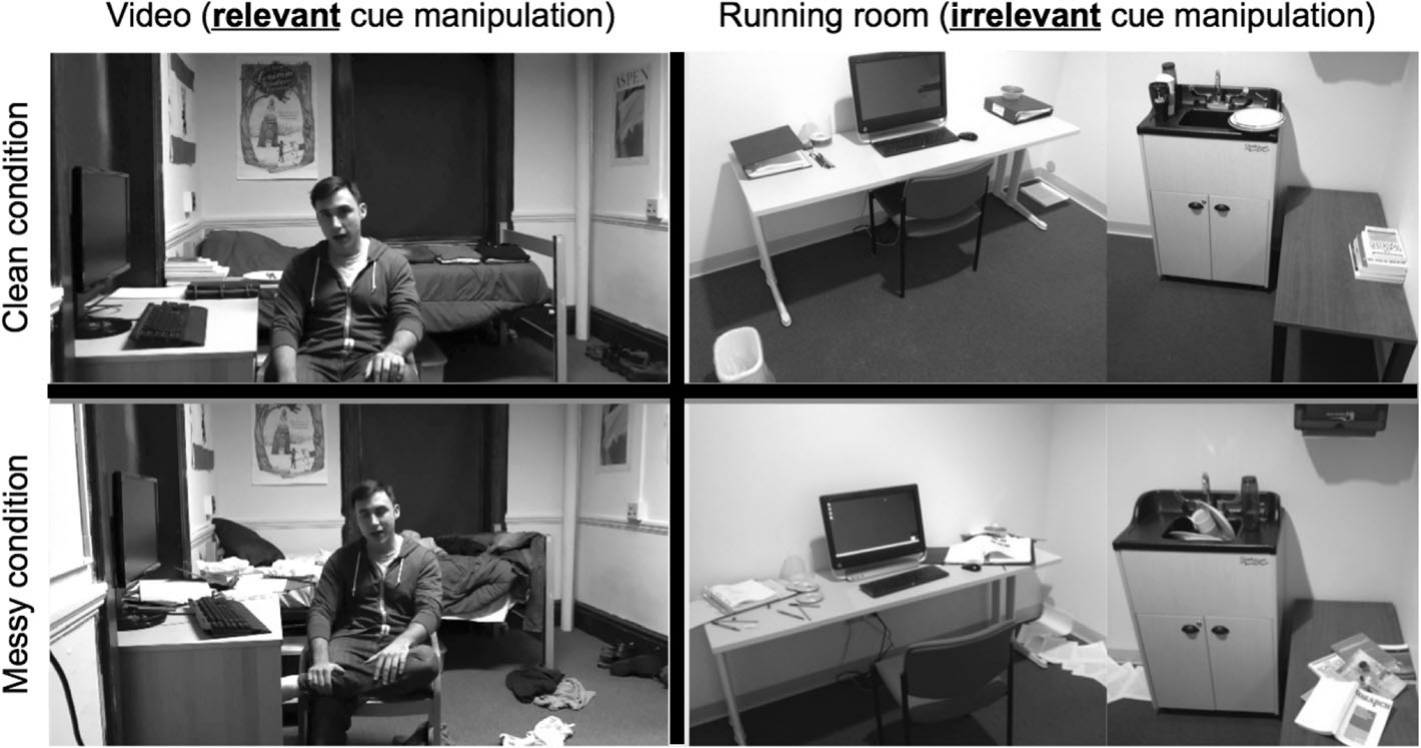
Screenshots depicting clean versus messy video conditions, representative of the relevant cue manipulation (left two quadrants), and testing room conditions, representative of the irrelevant cue manipulation (right two quadrants)

The study followed a two-by-two between-subjects design, with relevant cue (i.e., neat or messy arrangement of room in the video; described below) and irrelevant cue (i.e., neat or messy arrangements of the testing room; described below) as experimental factors, and participants’ media multitasking scores as a measured covariate. Accordingly, participants were randomly assigned to watch the video clip depicting either the neat or messy room (relevant cue), and also pseudo-randomly assigned to complete study tasks in either a neat or messy testing room (irrelevant cue). Since participants were often scheduled for the experiment back-to-back, the testing room was initially arranged in either a neat or messy configuration at the beginning of the day, and the testing room condition was subsequently counterbalanced by day. Props used in the messy/neat conditions for the testing room included cups, dishes, a stack of books, binders, pens, folders, a waste-paper basket, paper clips, binder clips, CDs, and miscellaneous papers (see Fig. 1).

After subjects provided informed consent, they were told they would be watching a video and completing several questionnaires. The experimenter brought the participant to the testing room, mentioning that “We are running a bit behind today and most of the rooms have been in use, so we’ll borrow this one.” This deception was necessary to prevent participants from becoming suspicious of the experimental manipulation of room, particularly the messy condition. The experimenter then explained that the participant would be watching a video of a Dartmouth student describing what he did yesterday, and that half of the interviews were pre-scheduled while the timing of the other half was a surprise. All subjects were told that the interview they would be watching was a surprise interview, to clarify that the target’s environment had not been preemptively cleaned or altered. Raters were led to believe that these video interviews were to be subsequently used for research on interview performance and corporate recruiting.

The video consisted of messy-background and neat-background versions (see Fig. 1, left two quadrants). A research confederate (who was not a Dartmouth undergraduate student, but one of the co-authors, Lopez) posed as an undergraduate student within the video, which was shot in a “student’s dormitory room” to provide a set of valid cues for trait analysis. The script was identical in both neat and messy conditions, and was designed to balance extraverted and introverted behaviors while reflecting the individual’s diverse interests. In this way, the confederate served as a neutral target who was difficult to evaluate based on non-verbal cues alone. For the messy and neat versions of the video respectively, clothes were either strewn across the bed and floor or folded neatly on the bed, the bed was unmade or made, and books and binders were either scattered across the bed and floor or on neat pile on the bed (see Fig. 1).

After viewing the video, subjects were brought into an adjacent room to complete a current state questionnaire, which was designed to provide a time buffer between viewing the video and rating the target. After completing the survey, participants were asked to “answer a few questions about the video.” Items on the trait inventory consisted of 11 conscientiousness items from the Big Five Inventory [16] interspersed with 7 filler items, also from the Big Five, representing traits orthogonal to conscientiousness (e.g., “has a lot of fun”). Participants indicated their responses to all items using a Likert scale ranging from 1 (not at all likely) to 5 (very likely). Several conscientiousness items were reverse-coded, so that a higher rating on any item reflected a greater endorsement of conscientiousness; following this reverse coding, all 11-items were summed to generate a composite conscientiousness score.

Next, participants completed a questionnaire assessing levels of media multitasking and distractibility. To assess media multitasking tendencies, we used the 18-item Media Multitasking-Revised (MMT-R) scale [6, 7], which has been developed and validated in another study using a large, independent sample (*N* = 995) and was found to have high internal reliability (Cronbach’s alpha = 0.86) [7]. As reported in [7], the MMT-R scale has a two-factor structure, with items loading on factors that reflect either: (1) (pro)active behaviors of compulsive/inappropriate phone checking, e.g., *“When talking to someone face-to-face, how often do you feel the urge to check your phone for unread messages, notifications?”*; or (2) more passive tendencies, including distractibility and interference from using various media, e.g., “*How often does your multimedia use interfere with your homework or work?*” Each scale item is answered on a 5-point likert scale, with the following response choices: 1-Never, 2-Rarely, 3-Sometimes, 4-Often, 5-Always for “how often” items, and 1-Not at all, 2, 3-Somewhat, 4, 5-Very(much) for all other items. There is one reverse-scored item, and all scale items (reflecting the abovementioned factors) are summed together, with a total possible score range of 18–90. Higher scores reflect a greater tendency to engage in MMT related behaviors.

At the conclusion of the experiment, all participants underwent a debriefing, were given the opportunity to ask any remaining questions, and reimbursed for their time with course credit.

### Manipulation check

As it was impossible to conduct a double-blind protocol with our video confederate, we conducted a follow up series of surveys to rule out behavioral confirmation effects as a potential confound. Both versions of the target video were cropped, removing background cues and leaving only the confederate’s face as a source of nonverbal information. Each version was then shown to an independent sample (*N* = 12) who completed the same series of trait ratings. We predicted that the ratings between video conditions would not differ significantly, in which case we could rule out the possibility that actual behavioral differences in the confederate contributed to differences in trait ratings. Independent *t*-tests confirmed that the confederate’s nonverbal cues did not differ significantly between versions of the video, all *p*’s > .05. Note, however, that although the confederate was not blind to room condition, this could not influence the results of the primary manipulation of interest (whether the testing room where the video was shown was neat or messy).

## Results

For our main analysis, we ran a multiple regression model with participants’ conscientiousness ratings of the target as the outcome variable, video (relevant cue) condition and room (irrelevant cue) condition as categorical predictors, and participants’ (centered) MMT scores as a continuous predictor. Unless otherwise stated, the alpha level for all inferential tests on parameter estimates from the model was set to .05. We also included several interaction terms, including: (1) the interaction between video (relevant cue) and room (irrelevant cue) conditions; (2) the interaction between MMT and the video (relevant cue) condition; and (3) the interaction between MMT and the room (irrelevant cue) condition (refer to Table 1 for complete model results and statistics).

**Table 1:** Parameter estimates from multiple regression model predicting participants’ conscientiousness ratings

			95% Confidence Interval			
Predictor	Estimate	SE	Lower	Upper	t	p
(Intercept)	37.785	0.5889	36.6152	38.9553	64.166	< .001
Room (irrelevant cue) condition	−1.210	1.1736	−3.5415	1.1223	−1.031	0.305
Video (relevant cue) condition	−7.828	1.1779	−10.1686	−5.4878	−6.646	< .001
Media multi-tasking (centered)	0.141	0.0658	0.0102	0.2716	2.142	0.035
Room Condition ✻ Media multi-tasking	−0.335	0.1317	−0.5963	− 0.0728	−2.540	0.013
Room Condition ✻ Video Condition	−0.264	2.3557	−4.9447	4.4169	−0.112	0.911
Video Condition ✻ Media multi-tasking	0.152	0.1316	−0.1097	0.4133	1.154	0.252

Overall, the model fit the data well, *F*(6, 89) = 9.22, *p* < .001, and captured a reasonable percentage of variance in conscientiousness scores, adjusted *R*^2^ = 0.342. Replicating prior research by Gosling and colleagues [11], there was a main effect of relevant cues (i.e., video condition) on participants’ conscientious judgments, with participants assigned to the messy (vs. clean) video condition providing lower conscientiousness ratings, *b* = − 7.83 (95% CI: -10.17, − 5.49), *t =* − 6.65, *p* < .001. There was no significant main effect of irrelevant cues (i.e., testing room condition), *p* = .305. There was, however, a main effect of media-multitasking, with those participants reporting frequent media multitasking providing higher conscientiousness ratings, regardless of testing room condition, *b* = 0.141 (95% CI: 0.01, 0.27), *t =* 2.14, *p* = .035, As far as the model’s interaction terms, there was a significant interaction between MMT and the room (irrelevant cue) condition, *b* = − 0.335 (95% CI: -0.60, − 0.07), *t =* − 2.54, *p* = .013.

**Fig. 2:**
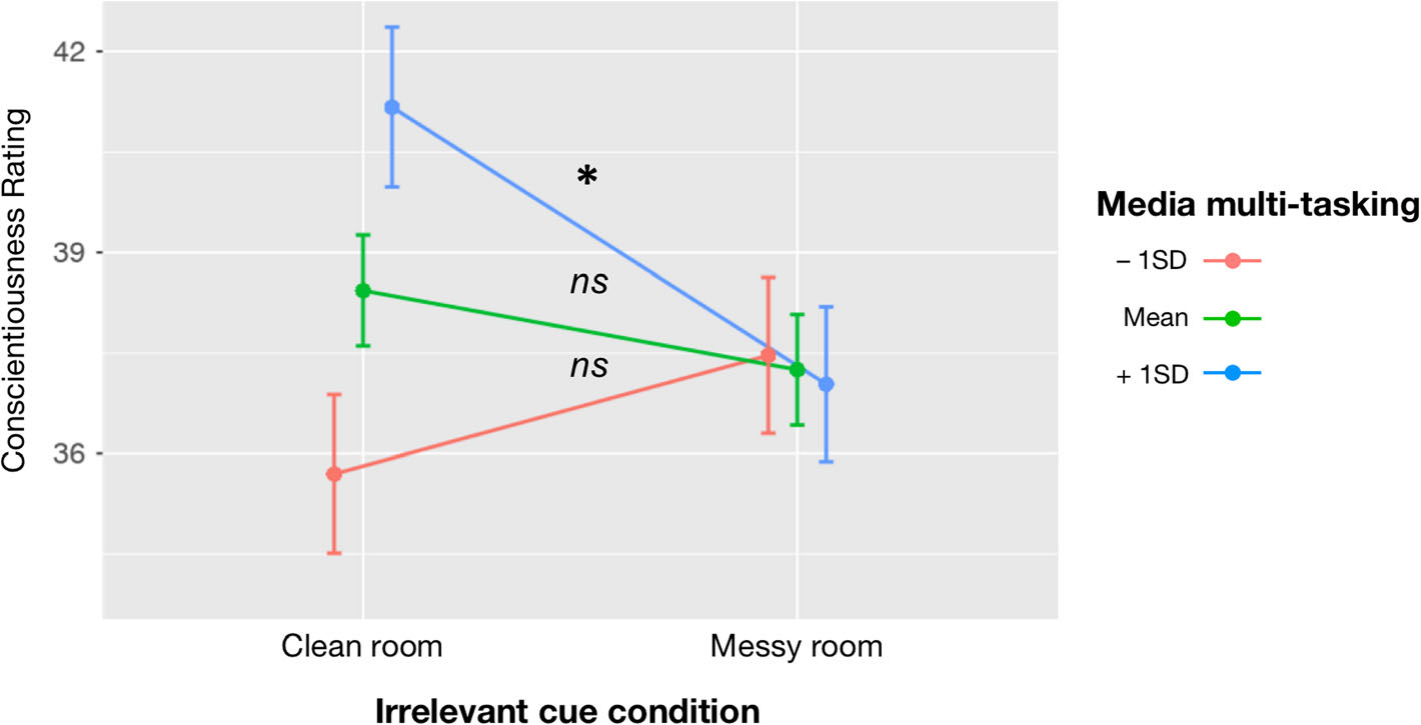
Line plot depicting simple slopes of the effect of room condition at different levels of media multitasking (MMT; as moderating variable), with –1SD MMT line in red, mean MMT line in green, and + 1SD MMT line in blue. Lines indicate standard error of the mean and asterisk indicates *p* < .05. Standard error bars are offset to avoid overlap

To unpack the interaction between MMT and room (irrelevant cue) condition, we ran simple slopes tests (with all other predictors held constant) to examine the effect of the room (irrelevant cue) condition at varying levels (i.e., − 1SD, mean, and + 1SD) of MMT. These tests revealed that among participants who reported low (-1SD; *N* = 16) or average levels of MMT (*N* = 66), there was no significant change in their conscientiousness judgments as a function of the room (irrelevant cue) condition, all *p*’s ≥ .282. But, for those reporting high (+1SD; *N* = 14) levels of MMT, there was a significant difference in their conscientiousness judgments, such that those assigned to view the video in the messy room condition made lower conscientiousness ratings of the target than those assigned to view the video in the neat room, *b =* − 4.22, *SE* = 1.67, *t* = − 2.53, *p* = .013 (See Fig. 2 for line plots depicting all simple slope effects) There was no significant interaction between the video (relevant cue) and room (irrelevant cue) conditions, *p* = .911, and no interaction between MMT and the video (relevant) condition, *p* = .252.

## Discussion

This study found that people who report frequent media multitasking are influenced by irrelevant cues during person perception. Specifically, the observed interaction between testing room condition and media multitasking suggests that media multitasking is associated with altered cue processing in the presence of incidental, irrelevant cues. High media multitaskers, compared to their low multitasking counterparts, readily incorporated incidental and irrelevant room cues into personality judgments of a social target’s overall conscientiousness. These findings suggest that media multitasking may characterize individuals who are more likely to be influenced by incidental environmental cues, and this person-by-environment interaction can impact subsequent perception and judgments of core personality traits, such as conscientiousness.

In addition to demonstrating a relationship between media multitasking and person perception, our study replicated and extended previous work showing that environmental cues affect person perception (e.g., [11]). First, the robust main effect of the relevant (i.e., video) cues on participants’ impressions of trait conscientiousness suggests that, in general, individuals tend to incorporate environmental cues into personality evaluation. Since participants were not explicitly directed to attend to the background in the video, we can presume that this cue incorporation potentially occurred automatically.

Given the nature of the effects we observed in the current study, we speculate that there may be implications of media multitasking behaviors in other domains, beyond person perception. For example, those who engage in more frequent media multitasking may have altered attentional processing that biases them to be more responsive toward other cues in the environment (e.g., food advertising) and/or emotionally evocative stimuli (e.g., physical danger or social threat). A potentially fruitful avenue of future work would be to examine the development of media multitasking behaviors in children and adolescents and assessing subsequent changes across domains, including person perception, appetitive behaviors, and emotional reactivity. Indeed, recent studies have begun employing longitudinal designs to address such questions, with one study showing that media multitasking and attentional problems not only co-vary, but early adolescents’ media multitasking tendencies may lead to increased distractibility over time [17]. Another study has demonstrated that some sub-groups of adolescents (i.e., middle school aged girls) are also prone to developing sleeping problems following increased media multitasking [18].

Despite the significance and novelty of our media multitasking finding for social cognition, there are some limitations in our study design and procedure that necessarily constrain the interpretability and generalizability of the results. First, although we experimentally manipulated multiple cue types (relevant and irrelevant), and participants were pseudo-randomly assigned to all conditions, the observed relationships with MMT are correlational. This precludes any strong inferences as to the directionality of the effects. For example, over time a person might become a high media multitasker, and this may induce changes in attentional processing of various cues. Or, a third variable (such as existing cognitive and attentional biases) may be a confounding factor, predisposing individuals to media-multitasking while also causing these individuals to more readily process extraneous cues—including irrelevant cues that could be incorporated into potentially inaccurate perception of others’ traits and qualities. If the former scenario is the case (i.e., increased MMT resulting in attentional biases over time), chronic media multitasking cannot be manipulated easily in short laboratory sessions. Of course, this is generally true of all research that examines behavior as a function of individual differences or personality. At best, future studies might consider employing longitudinal designs like those employed by Baumgartner and colleagues [17, 18] that measure people’s MMT tendencies and attentional processes at multiple time points, with a priori justification for an appropriate time scale and assessment intervals (see [19]).

Lastly, we did not incorporate additional measurements to track patterns of participants’ attention while they watched the video in the testing room. Thus, we cannot make strong claims as to the relative viewing time of room versus target cues for the video, as well as potentially switching attention between the video and the peripheral (irrelevant) neat or messy cues in the testing room. Future studies should address this by adding eye-tracking measures to this or a similar type of paradigm, and this would be illuminating to see whether high media multitaskers show distinct or divergent attentional profiles during person perception.

## Conclusions

To conclude, this study demonstrated that MMT is associated with altered processing of incidental, irrelevant cues that can impact person perception. The study design allowed us to investigate the role of incidental cues in the immediate environment that had greater or less relevance to guide person perception. Although the testing room cues were completely irrelevant to the task at hand, they nonetheless exerted effects on those participants with greater propensity to engage in media multitasking behaviors. This experimental design has ecological validity in that it simulates the type of common, multi-cue contexts in which people often find themselves, such as working on a computer in a neat (or messy) office setting, or doing housework while checking one’s phone. Future studies would benefit from using crossed designs like the one here, in which both relevant and irrelevant environmental cues are manipulated. This would enable researchers to examine whether media multitasking is associated with altered cue processing aspects of social cognition in which implicit processes are often at play, such as racial bias or stereotype formation. More broadly, the present work highlights subtle ways in which media multitasking behaviors are associated with altered perceptual processing of environmental cues.

## Abbreviations

MMT: Media multi-tasking
MMT-R: Media Multi-tasking Revised (scale)

## References

[1] Alvarez GA, Franconeri SL (2007). How many objects can you track?: evidence for a resource-limited attentive tracking mechanism. J Vis.

[2] Gazzaley A, Rosen LD. The distracted mind: ancient brains in a high-tech world. Cambridge: MIT Press; 2016.

[3] Ophir E, Nass C, Wagner AD (2009). Cognitive control in media multitaskers. Proc Natl Acad Sci.

[4] Cain MS, Mitroff SR (2011). Distractor filtering in media multitaskers. Perception.

[5] Lin L (2009). Breadth-biased versus focused cognitive control in media multitasking behaviors. Proc Natl Acad Sci U S A.

[6] Lopez RB, Heatherton TF. Media multitasking is associated with reduced activity in frontoparietal (versus reward) systems, lower trait self-control, and higher body mass index. Social cognitive and affective neuroscience annual meeting; 2017; Los Angeles, CA.

[7] Lopez RB, Heatherton TF, Wagner DD. Media multitasking is associated with higher risk for obesity and increased responsiveness to rewarding food stimuli. https://osf.io/qkgk4/. 10.1007/s11682-019-00056-0 30820857

[8] Heatherton TF (2011). Neuroscience of self and self-regulation. Annu Rev Psychol.

[9] Dijksterhuis A, Bargh JA. The perception-behavior expressway: automatic effects of social perception on social behavior. Adv Exp Soc Psychol Volume 33. Elsevier; 2001. pp. 1–40.

[10] Bargh JA, Morsella E (2008). The unconscious mind. Perspect Psychol Sci. Sage publications. Inc.

[11] Gosling SD, Ko SJ, Mannarelli T, Morris ME (2002). A room with a cue: personality judgments based on offices and bedrooms. J Pers Soc Psychol.

[12] Sadalla EK, Vershure B, Burroughs J (1987). Identity symbolism in housing. Environ Behav.

[13] Kay AC, Wheeler SC, Bargh JA, Ross L (2004). Material priming: the influence of mundane physical objects on situational construal and competitive behavioral choice. Organ Behav Hum Decis Process.

[14] Brunswik E. The conceptual framework of psychology. Chicago: University of Chicago Press; 1952.

[15] Cohen J (1988). Statistical power analysis for the behavioral sciences 2nd edition.

[16] John OP, Donahue EM, Kentle RL. Big five inventory [internet]. PsycTESTS Dataset.

[17] Baumgartner SE, van der Schuur WA, Lemmens JS, te Poel F (2018). The relationship between media multitasking and attention problems in adolescents: results of two longitudinal studies. Hum Commun Res.

[18] van der Schuur WA, Baumgartner SE, Sumter SR, Valkenburg PM (2018). Media multitasking and sleep problems: a longitudinal study among adolescents. Comput Human Behav.

[19] Kom EL, Graubard BI, Midthune D (1997). Time-to-event analysis of longitudinal follow-up of a survey: choice of the time-scale. Am J Epidemiol.

